# A Contemporary Review of Subcutaneous and Extravascular Implantable Cardiac Defibrillators

**DOI:** 10.19102/icrm.2026.17055

**Published:** 2026-05-15

**Authors:** Ahmad Jalil, Wajeeha Zahid, Fatima Rajab, Mahmoud Gomaa, Ahsan Sakef, Muhammad R. Afzal

**Affiliations:** 1 Department of Internal Medicine, Baptist Memorial Hospital-North Mississippi, Oxford, MS, USA; 2 Department of Internal Medicine, University of Arkansas Little Rock, Little Rock, AR, USA; 3 King Edward Medical University, Lahore, Pakistan; 4 Division of Cardiovascular Medicine, The Ohio State University Wexner Medical Center, Columbus, OH, USA; 5 The Ohio State University College of Medicine, Columbus, OH, USA

**Keywords:** Cardiac arrhythmia, extravascular ICD, implantable cardioverter-defibrillator, subcutaneous ICD, sudden cardiac death

## Abstract

Implantable cardioverter-defibrillators (ICDs) are life-saving devices used to prevent sudden cardiac death. Two newer types of ICDs—the extravascular ICD (EV-ICD) and the subcutaneous ICD (S-ICD)—offer alternatives to traditional transvenous ICDs. Additionally, efforts are being made to introduce defibrillation and pacing interventions through a single device called the modular cardiac rhythm management system (mCRM). This review compares the EV-ICD and S-ICD based on currently available clinical data, focusing on their safety and performance. Additionally, we review modular ICD systems, which integrate leadless pacing with defibrillation. We conducted a comprehensive literature search of PubMed and ScienceDirect, focusing on articles demonstrating the clinical outcomes of EV-ICDs and S-ICDs as well as modular systems. However, we found that sufficient studies are not available to determine the clinical efficacy and safety outcomes of EV-ICDs and the mCRM. Additionally, comparative studies between EV-ICDs and S-ICDs are still required to determine their relative roles in the field of electrophysiology, with the goal of empowering clinicians to understand these emerging technologies to support better patient care decision-making.

## Introduction

The implantable cardioverter-defibrillator (ICD) is one of the most important devices used to prevent sudden cardiac death. ICDs are used in high-risk cases, including in patients with sustained ventricular tachyarrhythmias, survivors of sudden cardiac arrest without reversible causes, patients with chronic systolic heart failure, and also those with certain inherited arrhythmia syndromes.^1^

Since their introduction in 1980, many large studies have shown that ICDs improve survival compared with anti-arrhythmic drugs.^2,3^ Despite their effectiveness, however, ICDs are associated with risks and complications that can occur early, such as lead dislodgement, lead fracture, vascular injury, pneumothorax, cardiac perforation, pericardial effusion, and tamponade Moreover, potential chronic issues include systemic infection, insulation failure, and conductor fracture, leading to inappropriate shocks or device malfunction. Lead failure remains the most common problem, and many patients outlive their leads.^4–8^

These issues led to the development of less-invasive options such as the subcutaneous ICD (S-ICD), which avoids the vascular system entirely. The S-ICD (EMBLEM MRI S-ICD™ system; Boston Scientific, Marlborough, MA, USA) is implanted entirely subcutaneously, avoiding contact with cardiac or vascular structures. Early versions lacked anti-tachycardia pacing (ATP) and remote monitoring but effectively detected and treated ventricular arrhythmias.^9–11^

More recently, the extravascular ICD (EV-ICD) (Aurora EV-ICD™ system; Medtronic, Minneapolis, MN, USA) was introduced to overcome these limitations, offering a smaller generator and ATP with asystole pacing features. It provides a complete option for patients needing both pacing and defibrillation while avoiding transvenous risks.^12–14^

This review compares the EV-ICD and S-ICD based on current data, focusing on their safety, performance, and integration with leadless pacing (LP) systems.

## Clinical significance of implantable cardioverter-defibrillator therapy

ICDs are state-of-the-art devices designed to perform a range of functions from cardiac monitoring and sensing to arrhythmia detection and therapeutic interventions, providing survival benefits in a wide variety of patients, especially survivors of cardiac arrest.^15–17^ They are known to provide a clinical benefit in patients with a wide variety of conditions, including previous myocardial infarction, ventricular fibrillation (VF), ventricular tachycardia (VT), Brugada syndrome, long QT syndrome, and hypertrophic cardiomyopathy.^15–18^

The ICD uses digital signal-processing algorithms to detect signals from the heart and interpret them, thereby enabling cardiac sensing and arrhythmia detection. Rate and rhythm detection are two crucial components for proper therapeutic intervention by ICDs. Three detection categories include sinus rhythm, VF, and VT. Similarly, supraventricular tachycardia/VT discrimination, a crucial aspect of therapy, is where rhythm detection comes into action.^15^ Therapeutic interventions that are possible with ICDs include: (1) defibrillation with high-energy shocks (unsynchronized), (2) cardioversion with low-energy shocks (synchronized), and (3) ATP. Bradycardia pacing, another new intervention similar to conventional pacemakers, is a feature of some types of ICDs.^15,16^

## The progression of implantable cardioverter-defibrillators: the path to extravascular and modular implantable devices

The evolution of ICDs has progressed through a journey of transformation since the first device was developed by Mirowski and colleagues to balance the clinical efficacy and the long-term complications of different types of these devices.^19,20^

Transvenous ICDs (TV-ICDs) were the first widely used systems, and, due to their intracardiac lead location, they are associated with lead-related complications and infection risk. To reduce these risks, S-ICDs were developed; they are implanted entirely subcutaneously, resulting in fewer lead-related and vascular complications.^21^ However, S-ICDs lack some features, such as ATP and bradycardia pacing.^22^

To address these functional limitations while still avoiding transvenous leads, EV-ICDs were introduced; they can deliver ATP, with a reported termination rate of ~70% of ventricular arrhythmic episodes.^23–25^ A novel approach involving wireless intrabody communication between devices has been used in a modular cardiac rhythm management (mCRM) system, consisting of a communicating ATP-enabled leadless cardiac pacemaker (LCP) and an S-ICD.^24^

## Indications and contraindications

According to the American College of Cardiology, some ICD indications are:

- ***Primary prevention***: For patients with a left ventricular ejection fraction (LVEF) of ≤35% with New York Heart Association (NYHA) functional class II or III symptoms and patients with LVEF ≤ 30% following a previous myocardial infarction.^16,26^
- ***Secondary prevention***: For patients who have experienced sudden cardiac arrest due to unstable VT or VF without any reversible cause, as well as those with spontaneous sustained VT associated with structural heart disease.^16,26^

Contraindications include VT/VF with a reversible cause, VT/VF amenable to surgical or catheter ablation, incessant VT/VF, and patients with drug-refractory NYHA class IV congestive heart failure who are not candidates for cardiac resynchronization therapy or heart transplantation.^15,26^

## Extravascular and subcutaneous implantable cardioverter-defibrillators: next-generation devices

The anatomical locations of EV-ICDs and S-ICDs—outside the heart in substernal and subcutaneous positions—set them apart from TV-ICDs in terms of reducing the risk of complications such as infections.^21–28^ Moreover, the clinical efficacy of these devices, particularly S-ICDs, is comparable to that of TV-ICDs.^29,30^ However, the absence of ATP makes them less useful in certain scenarios compared to TV-ICDs.^21,22^ EV-ICDs incorporate useful features from both S-ICDs and TV-ICDs, offering ATP as well as an extracardiac location with a lower risk of complications.^23^ Additionally, their defibrillation thresholds, smaller size, and longer projected battery longevity make them a suitable choice for many patients.^23,25^

## Differences between extravascular and subcutaneous implantable cardioverter-defibrillators

Here, we present a brief comparative review of the differences between EV-ICDs and S-ICDs. However, clinical studies or trials directly comparing the efficacy and safety of these devices are not available.

### Lead position and anatomic consideration

The EV-ICD positions its lead in the substernal space, closer to the myocardium, while the S-ICD lead is placed subcutaneously above the sternum.^23,27,28^ While still avoiding the vascular space, the deeper EV-ICD lead position introduces different procedural considerations, including substernal dissection and proximity to mediastinal structures, factors that are all directly impacted by anatomic variations. This difference in anatomical position also allows lower energy requirements by EV-ICD, leading to a smaller generator size and 60% longer battery life as compared to S-ICDs. Additionally, this enhanced durability also leads to a decrease in the number of battery replacements in EV-ICD patients compared to S-ICD patients.^23–25^

### Efficacy and pacing capabilities

When it comes to terminating arrhythmias, both devices work effectively. A study by Friedman et al. demonstrated that the rate of successful defibrillation by EV-ICDs at implantation was 98.7%, and the rate at 6 months was similar to that of S-ICDs. However, the first shock efficacy for the resolution of discrete spontaneous arrhythmic episodes of EV-ICDs was 78%, which is lower than that of S-ICDs.^23,25^ The ability of EV-ICDs to deliver pacing, including ATP and backup pacing for slow cardiac rhythms, is one significant distinction. Patients who would previously need a transvenous system are now more eligible, especially those who have recurring ventricular tachyarrhythmias that can be terminated with pacing. In contrast, S-ICDs cannot provide any pacing support.^21–23^ As a result, patients with pacing indications or frequent monomorphic VT may experience higher rates of shock therapy.

### Safety profile and long-term consideration

Both EV-ICDs and S-ICDs have favorable safety profiles:

- A pivotal study of EV-ICDs demonstrated 91.9% and 89% rates of freedom from system- and procedure-related complications at 1 and 3 years, respectively, comparable to those of S-ICDs, which demonstrated a rate of 88.6% in a study by Brouwer et al.^21,29,31^
- Both devices have comparable rates of device removal due to infections.^16,18^ Brouwer et al. reported that 2.6% of S-ICD patients required invasive intervention due to infection.^21^ Similarly, EV-ICDs were associated with infections in 2.5% of patients in another study.^29^ Also, EV-ICDs are associated with a low risk of lead-related complications like S-ICDs.^21^

While reviewing the procedure times, an expert analysis revealed that the procedure times for EV-ICD implants are similar to early S-ICD procedures.^25^ However, EV-ICD implantation has a learning curve, which requires collaboration between electrophysiologists and cardiac surgeons. Early EV-ICD studies suggest acceptable safety outcomes, though long-term data remain limited compared with S-ICD experiences. Substernal lead placement introduces novel procedural risks that warrant careful patient selection and operator expertise. Moreover, the use of EV-ICDs is also associated with some restrictions—implantation in those with prior sternotomy, chest radiation, pericardial disease, or mediastinitis is contraindicated—while S-ICDs have fewer anatomical exclusions.^25^ Ongoing post-market surveillance will be critical in defining long-term complication rates.

### Sensing challenges, inappropriate therapies, and special features

Both EV-ICDs and S-ICDs face challenges with inappropriate therapies. EV-ICDs are associated with an inappropriate shock rate of 9.8% at 1 year, with P-wave oversensing representing the most common cause.^31^ S-ICDs are also associated with inappropriate shocks, often mistaking T-waves for dangerous rhythms, leading to unnecessary shocks.^31,33^

Several new systems, such as the SMART Pass filter (Boston Scientific), can help reduce inappropriate shock rates in people with S-ICDs, with one study of 1984 patients reporting >50% reduction in the risk of all inappropriate shocks.^34^

Meanwhile, inappropriate therapy with EV-ICDs can be reduced by lead positioning. In a study of 1,984 patients, SMART Pass reduced the risk of inappropriate shock by > 50%. Moreover, the P-wave sensing algorithm is a novel feature that can be incorporated into EV-ICDs. It identifies accurate rhythms by identifying alternating high amplitudes of R-waves and low amplitudes of P-waves.^25,35^

### Quality of life and cost-effectiveness

A study by Sears et al. compared the quality of life in patients with EV-ICDs to that reported in studies using other systems and found favorable outcomes.^36^ The longer battery life of EV-ICDs leads to fewer replacements and decreases the total cost for patients, thereby improving their quality of life.^31^ On the contrary, S-ICDs were associated with higher pain scores compared to TV-ICDs in the ATLAS (“Avoid Transvenous Leads in Appropriate Subjects”) trial, although pain decreased by 6 months.^37^ Another key point is that S-ICD implantation requires a special electrocardiogram screening beforehand to ensure the device senses signals properly.^38^

## Patient selection criteria

Non–TV-ICDs are used in patients with an indication for either secondary prevention or primary prevention plus a contraindication to TV-ICDs, such as:

- A reason for preservation of vascular access (younger age, chronic dialysis);
- Lack of vascular access; or
- A previous history of a complication of a TV-ICD leading to its explanation.

S-ICDs are indicated if patients fulfil the aforementioned criteria plus:

- Having no requirement for ATP for ventricular arrhythmias or
- Having no requirement for resynchronization therapy or anti-bradycardia pacing.^39^

EV-ICDs can be used in such conditions requiring ATP.^23^

## Clinical efficacy and safety

Several clinical trials to date have evaluated the efficacy and safety of EV-ICDs and S-ICDs.

### Performance outcomes of extravascular implantable cardioverter-defibrillators in clinical trials

Friedman et al. conducted a randomized controlled trial called the EV-ICD Pivotal Study, a pre-market study, to determine long-term device safety and performance outcomes in 316 patients with EV-ICDs **(Table 1)**. The study reported a 100% success rate for shock therapy, with a 17.5% inappropriate shock rate recorded at 3 years mainly attributed to P-wave oversensing. Additionally, it also revealed that ATP was successful in 77.1% of episodes, with increased usage at the end of follow-up and no major intraprocedural complications. Some major complications during follow-up included lead dislodgement in 2.8% of patients and infection in only 2.5% of patients with EV-ICDs.^23,31^ Similarly, Crozier et al. demonstrated stable pacing, sensing, and defibrillation performance of EV-ICDs in a small cohort of patients over a 3-year follow-up period.^40,41^

**Table 1:** Characteristics and Outcomes of Studies Using EV-ICD as an Intervention

Study	Design	Population (n)	Intervention	Outcomes	Key Results	Notes
Friedman et al. (2025)^31^	Pre-market, nonrandomized trial	316	EV-ICD	Defibrillation efficacy; ATP performance; safety profile; inappropriate therapy	100% shock therapy efficacy; 17.5% inappropriate shock rate at 3 years; 77.1% ATP success; 89% freedom from major complications	High defibrillation efficacy; effective ATP; few adverse effects
Swerdlow et al. (2024)^32^	Prospective, multicenter, nonrandomized pivotal study	299	EV-ICD with substernal lead	VF/VT detection performance; oversensing; inappropriate therapies	100% VF detection; 10.4% inappropriate therapy rate at 1 year; myopotentials and P-wave oversensing were the most common causes	Confirms feasibility and highlights areas for sensing optimization
Crozier et al. (2023)^41^	Retrospective study	14	EV-ICD	Inappropriate therapy; adverse effects	14 patients; 6.9% inappropriate shock rate; 21.4% had lead-related adverse effects	Small sample size
Chan et al. (2017)^42^	Prospective, nonrandomized study	16	Substernal-lateral coil with skin patch (ex vivo)	Substernal defibrillation success (35 J); safety; procedural feasibility	92.9% defibrillation success (13/14 patients); three procedure-related adverse events; one failure due to improper coil position	First-in-human feasibility of substernal ICD shocks; emphasized proper defibrillation lead position
Molnár et al. (2022)^43^	Retrospective analysis	45	Substernal lead implantation EV-ICD system	Defibrillation success; adverse events	90% defibrillation success (≥10 J margin); very few adverse events (1 patient); larger rib cage and posterior heart position linked to failures	First anatomical mapping of substernal space for ICD implantation

*Abbreviations:* ATP, anti-tachycardia pacing; EV-ICD, extravascular implantable cardioverter-defibrillator; ICD, implantable cardioverter-defibrillator; VF, ventricular fibrillation; VT, ventricular tachycardia.

Molnár et al. and Chan et al. also reported on the performance of substernal ICDs and lateral electrodes, respectively, with successful defibrillation documented in nearly 90% of patients.^42,43^

Enlighten, the EV-ICD post-approval registry, is sponsored by Medtronic. It is a study conducted within Medtronic’s post-market surveillance platform. Preliminary results from this study have been gathered from 228 patients, with successful defibrillation testing completed in 99%. Additionally, tunneling and lead placement were also successful in 96.9% of patients, with a 3.9% complication rate at discharge.^44,45^

### Performance outcomes of subcutaneous implantable cardioverter-defibrillators in clinical trials

Multiple clinical trials have reported the efficacy and safety of S-ICDs, covering inappropriate shock rates, infection, lead-related complications, and mortality **(Table 2)**. Brouwer et al. conducted a study to determine and compare the efficacy and safety of S-ICDs with TV-ICDs and demonstrated a first shock conversion efficacy of 88.6% and an 11.9% inappropriate shock rate. All-cause complications were experienced by 9% of patients, with a 93.7% survival rate in the S-ICD group.^29^ Additional comparative studies have reported broadly similar outcomes between S-ICDs and TV-ICDs.^27–30^ A study by Schiavone et al. compared the outcomes of S-ICDs between men and women and concluded that women are less likely to experience appropriate therapy, while no such correlation existed for device-related complications.^46^ Post-approval studies have also reported a shock efficacy of 98.4% and a complication rate of 13.5% at 5 years.^48,49^

**Table 2:** Characteristics and Outcomes of Studies with S-ICD as an Intervention

Study	Type of Study	Number of Participants	Mean Age (Years)	Duration	Outcomes	Complications
Weiss et al. (2013)^9^	Prospective nonrandomized multicenter	330 enrolled; 314 implanted	52 ± 16	11 months	180-day complication-free rate, 99%; VF conversion >90%; all VT/VF converted	13.1% IAS; no major perioperative issues
Boersma et al. (2017, EFFORTLESS)^14^	Observational registry	985	48 ± 17	3.1 years	Appropriate shocks, 13.5%; IAS, 11.7%; conversion, 97.4%	Complications, 2%; infection, 2.4%; erosion, 1.7%
Brouwer et al. (2016)^21^	Retrospective study	140 in the S-ICD group	41 (26–52)	5 years	17% appropriate therapy; inappropriate in 20 patients	4.1% infection; 0.8% lead complications
Knops et al. (2020)^27^	Clinical trial	426 in the S-ICD group	63 (54–69)	49.1 months	Appropriate shocks, 19.2%; IAS, 9.7%	4 infections; 5 lead-related complications
Brouwer et al. (2018)^29^	Comparative analysis	798 in the S-ICD group	49 ± 16	3 years	Appropriate shocks, 9.9%; IAS, 11.9%	21 infections; 5 lead-related complications
Kobe et al. (2013)^30^	Case–control study	69 in the S-ICD group	46 ± 16	217 days	VF conversion rate, 89.5%; inappropriate episodes in 5.2% due to T-wave oversensing	1 infection
Schiavone et al. (2024)^46^	Clinical trial	374 men; 374 women	48.8 (F); 47.8 (M)	26.5 months	Appropriate shocks, 7% (F) and 11.2% (M); inappropriate, 8.6% (F) and 11.5% (M). Sex is not a predictor of IAS or device-related complications	Minor lead issues
Gold et al. (2021)^49^	Prospective multinational cohort	1111	55.8 ± 12.4	18 months	IAS-free, 95.9%; shock-free, 94.3%; conversion success, 98.4%	Infections, 1.1%; 12 explants due to infection, syncope in 10 patients
Gold et al. (2023)^48^	Prospective multicenter registry (post-approval)	1643	53 ± 15	4.2 years	Shock efficacy, 98.4%; first shock, 92.2%; IAS, 15.8%	5-year complications, 13.5%; infections, 2.8%
Mithani et al. (2018)^51^	Cohort study	91 in the S-ICD group	55 ± 14	6 months	IAS, 1.1%	3.3% infections requiring explant
Liang et al. (2019)^52^	Comparative study	86 in the S-ICD group	45	23 months	Appropriate therapy, 1.2%; inappropriate therapy, 9.3%	1 infection
Viani et al. (2019)^53^	Clinical study	90 in the S-ICD group	53 ± 13	17 months	Effective defibrillation testing in all patients; inappropriate therapy in 5	1 infection; 2 lead-related complications
Olde Nordkamp et al. (2024)^54^	Randomized controlled trial	426 in the S-ICD group	63 (54–69)	49.1 months	9.9% cumulative inappropriate therapy	N/A
Rella et al. (2024)^55^	Observational study	70 in the S-ICD group	N/A	5 ± 2.3 years	4 inappropriate therapies; 2 due to T-wave oversensing	1 lead-related complication
Healey et al. (2022)^56^	Randomized clinical trial	251 in the S-ICD group	48	6 months	Successful defibrillation testing in 96.2%; IAS, 6.4% (2.7%/year)	0.4% lead-related complications
Friedman et al. (2016)^57^	Retrospective analysis	3717 in the S-ICD group	53.5	N/A	High defibrillation success	3 infections; 5 lead complications

*Abbreviations:* F, female; IAS, inappropriate shock; M, male; N/A, not available; S-ICD, subcutaneous implantable cardioverter-defibrillator; VF, ventricular fibrillation; VT, ventricular tachycardia.

### Defibrillation testing and complications

The success of defibrillation testing with S-ICDs has been consistently high across studies, with reported conversion rates ranging from 89.5%–98.4% in multicenter trials; the EFFORTLESS trial reported a 97.4% conversion rate for spontaneous episodes.^14,48^ Complication rates have varied, with infection rates ranging from 2.8% to as high as 4.1%.^21,48^ However, lead-related complications have been low—for example, just 0.4% in a study by Healey et al.—depending on the study size and follow-up duration.^56^ In contrast, however, the incidence of inappropriate shocks was relatively high, exceeding 11% in the EFFORTLESS trial.^14^ Overall, S-ICDs present a high defibrillation success rate (≥90%) with generally acceptable complication rates, reinforcing their safety and efficacy across diverse patient populations. Similarly, EV-ICDs also offer nearly 90% defibrillation success with an ATP success rate of >70%; however, the rate of inappropriate therapy has also been high (>10%).^42,43^

## Modular cardiac rhythm management

**Figure 1:**
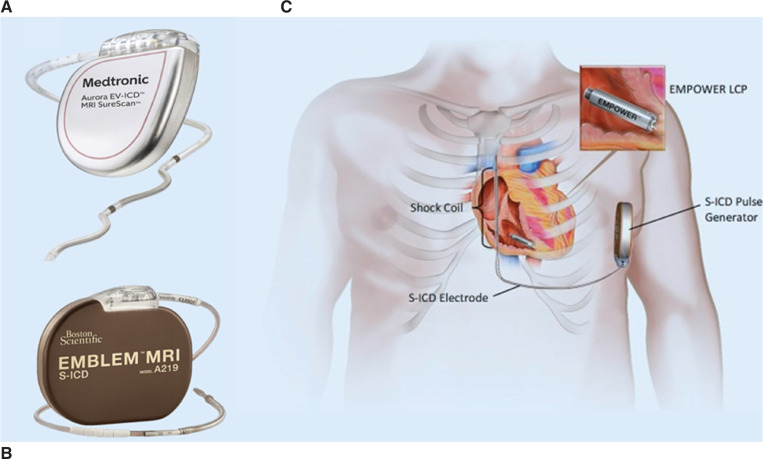
Visual illustration of **(A)** Medtronic’s Aurora™ extravascular implantable cardioverter-defibrillator^47^ and **(B)** Boston Scientific’s EMBLEM™ MRI subcutaneous implantable cardioverter-defibrillator^50^ and **(C)** modular cardiac rhythm management system. *Abbreviation:* LCP, leadless cardiac pacemaker; S-ICD, subcutaneous implantabl cardioverter-defibrillator.

The mCRM system is a novel approach involving wireless intrabody communication between devices, consisting of an ATP-capable LCP and an S-ICD **(Figure 1)**.^24^

### Device–device communication in modular cardiac rhythm management

Within the mCRM system, the LCP senses the signals sent by the S-ICD via its anode and cathode in the same manner it senses intrinsic cardiac pacing. To ensure reliable communication between the S-ICD and LCP, interdevice interaction demonstrates three features: (1) redundancy of two back-to-back messages, (2) coupling of communication to sensed R-waves, and (3) low-amplitude voltage with a 25-kHz frequency of emitted signals.^24^

### Efficacy and safety of modular cardiac rhythm management in clinical trials

Preclinical studies have shown adequate S-ICD sensing during normal sinus rhythm, LP, and VT/VF with successful communication between devices.^22,58,59^ Ahmed et al. demonstrated the successful use of the Micra™ LP (Medtronic) and S-ICD therapy in an 81-year-old man, including simultaneous device–programmer communication, successful S-ICD rhythm discrimination during LP communication and pacing, and normal post-shock LP performance with effective defibrillation.^22^

The MODULAR ATP (“Effectiveness of the EMPOWER™ Modular Pacing System and EMBLEM™ Subcutaneous ICD to Communicate Antitachycardia Pacing”) trial is a global clinical trial designed to evaluate the efficacy of the first intercommunicating LP system (EMPOWER™ LP system; Boston Scientific) with the S-ICD (EMBLEM™ S-ICD), including validation of device-to-device communication, pacing performance, rate responsiveness, and short-term system safety.^60^ The first 6-month results of the clinical trial showed that this device met all the safety and efficacy endpoints. The study demonstrated an excellent communication rate of 98.8% between devices, with an ATP success rate of 61.3%. It also demonstrated an excellent safety profile within the first 6 months of its use, with a major complication-free rate of 97.5% and no patient requests for deactivation due to pain or discomfort. Additionally, the pacing capture threshold of ≤2.0 V at 0.4 ms was present in 97.4% of patients.^60,61^

## Food and Drug Administration approval and implantable cardioverter-defibrillators

Among ICDs, both S-ICDs and EV-ICDs have been approved by the US Food and Drug Administration (FDA).^62,63^ However, Boston Scientific is currently developing the mCRM™ system, which pairs the EMBLEM™ MRI S-ICD with the investigational EMPOWER™ LP system; the company has stated that FDA approval is being pursued.^64^ The Medtronic Aurora™ EV-ICD was approved by the FDA in 2023 and is available in the US market. It was approved in light of findings from the EV-ICD Pivotal Study. Boston Scientific’s EMBLEM™ MRI S-ICD System was approved by the FDA in 2016. It introduced two new features, including SMART Pass technology and the Atrial Fibrillation Monitor™.^63^ The SMART Pass technology increases the accuracy of the INSIGHT™ algorithm and helps prevent the delivery of inappropriate shocks to patients. Similarly, the Atrial Fibrillation Monitor™ helps identify atrial fibrillation and alerts physicians, thereby supporting better decision-making.^34,63^

## Current barriers and challenges

Several factors currently limit the broader adoption of EV-ICDs. EV-ICDs cannot be used in certain cases, including in patients who require cardiac resynchronization therapy or chronic bradycardia pacing. Other patient candidate restrictions include those with prior sternotomy, chest radiation, pericardial disease, or mediastinitis. Lastly, as the device is not widely available, it is associated with a steep learning curve, which requires close collaboration between cardiac surgeons and electrophysiologists.^25^

## Future directions and advancements

Several investigations are being carried out to improve existing EV-ICD options. One of these advancements is the introduction of a parasternal DF4 lead in the anterior mediastinum close to the pericardium, which can help in availing the benefits of both EV- and TV-ICDs by connecting to the transvenous generators placed in the pectoral area.^65^ One study documented the safety and reliability of placing a novel EV-ICD with effective defibrillation and sensing of abnormal heart rhythms using DF-4 ICD pulse generators.^65^

In addition, the development of modular systems, introduction of new and advanced algorithms, and the adoption of patient-tailored approaches can redefine the use of ICDs for a broader and complex population.^24,34,35^ The next generation of mCRM systems is likely to incorporate leadless devices designed to pace multiple heart chambers. With these enhancements, dual-chamber pacing and cardiac resynchronization therapy could be achieved either independently or in tandem with an S-ICD implanted alongside them.^24^ Lastly, machine learning and artificial intelligence could play a promising role in rhythm classification and reducing inappropriate shocks, thus improving both the quality of life and the quality of care.^66^

The comparison between the S-ICD and EV-ICD is summarized in **Table 3**, which was developed using insights from a recent expert debate on the topic.^67^

**Table 3:** Comparison Between EV-ICD and S-ICD^67^

Parameter	EV-ICD	S-ICD
Design and capabilities	Substernal lead allowing defibrillation and pacing including ATP, asystole support, and pause-prevention pacing	Subcutaneous lead for defibrillation only; no chronic pacing unless paired with LLPM (EMPOWER™ modular system)
DFT	DFT success at implant, 98.7%	DFT success at implant, 97%
ATP efficacy	77%, comparable to transvenous systems	61% in the S-ICD–LLPM modular system study
Capture threshold	5.09 V at 0.5 ms (higher and less efficient)	≤2 V at 0.4 ms in 97% of cases (better efficiency)
Implantation success	95% (299/316 cases)	100% (292/292 cases)
Anesthesia requirement	Requires general anesthesia	Can be performed without general anesthesia
Fluoroscopy and Imaging	Requires fluoroscopy and often chest CT imaging for substernal lead tunneling	No fluoroscopy or CT imaging required; guided by anatomical landmarks
VF induction and defibrillation testing	Typically performed	Not required
Procedural complexity	More complex and requires hybrid lab setup	Simpler procedure that can be performed in a standard electrophysiology lab with minimal setup
Size	Approximately half the size of the S-ICD	Larger generator compared with EV-ICD
Complication rate at 6 months	7.4%	4.1%
Major complications at 1–3 years	9.2% major complications, mainly lead dislodgement; infection, 4.7%; inappropriate shocks, 9.8% at 1 year and 17.5% at 3 years; system revisions, 7.6%	Lower overall complication and infection rates; SMART Pass filter significantly reduces inappropriate shocks
Pacing interface	Substernal electrode in epicardial fat, leading to higher pacing thresholds	EMPOWER™ pacemaker has direct myocardial contact, resulting in better thresholds

*Abbreviations:* ATP, anti-tachycardia pacing; CT, computed tomography; DFT, defibrillation threshold; EV-ICD, extravascular implantable cardioverter-defibrillator; LLPM, leadless pacemaker; S-ICD, subcutaneous implantable cardioverter-defibrillator; VF, ventricular fibrillation.

## Conclusion

S-ICDs provide effective defibrillation while avoiding TV lead–related complications, but they lack ATP and chronic bradycardia pacing. The EV-ICD, offering both pacing and defibrillation without TV leads, is a promising new addition to ICD therapy and may expand non-TV options for selected patients. Modular systems such as mCRM are also emerging to integrate defibrillation with LP, but longer-term data remain limited. Further comparative studies of EV-ICD and S-ICD devices are needed to refine patient selection and define their roles in practice.
